# Elementary calcium signaling in arterial smooth muscle

**DOI:** 10.1080/19336950.2019.1688910

**Published:** 2019-12-04

**Authors:** Gang Fan, Yingqiu Cui, Maik Gollasch, Mario Kassmann

**Affiliations:** a Charité – Universitätsmedizin Berlin, Experimental and Clinical Research Center (ECRC), a joint cooperation between the Charité Medical Faculty and the Max Delbrück Center for Molecular Medicine (MDC), Berlin, Germany; b DZHK (German Centre for Cardiovascular Research), partner site Berlin, Germany

**Keywords:** Ca^2+^ sparks, ryanodine receptors, Ca_v_1.2, Ca_v_3.2, caveolae

## Abstract

Vascular smooth muscle cells (VSMCs) of small peripheral arteries contribute to blood pressure control by adapting their contractile state. These adaptations depend on the VSMC cytosolic Ca^2+^ concentration, regulated by complex local elementary Ca^2+^ signaling pathways. Ca^2+^ sparks represent local, transient, rapid calcium release events from a cluster of ryanodine receptors (RyRs) in the sarcoplasmic reticulum. In arterial SMCs, Ca^2+^ sparks activate nearby calcium-dependent potassium channels, cause membrane hyperpolarization and thus decrease the global intracellular [Ca^2+^] to oppose vasoconstriction. Arterial SMC Ca_v_1.2 L-type channels regulate intracellular calcium stores content, which in turn modulates calcium efflux through RyRs. Ca_v_3.2 T-type channels contribute to a minor extend to Ca^2+^ spark generation in certain types of arteries. Their localization within cell membrane caveolae is essential. We summarize present data on local elementary calcium signaling (Ca^2+^ sparks) in arterial SMCs with focus on RyR isoforms, large-conductance calcium-dependent potassium (BK_Ca_) channels, and cell membrane-bound calcium channels (Ca_v_1.2 and Ca_v_3.2), particularly in caveolar microdomains.

## Introduction

Hypertension is the major risk factor for cardiovascular disease (stroke, myocardial infarction, and others) – the most common cause of death worldwide. A main contributor to systemic blood pressure regulation is the resistance of small arteries and arterioles in the peripheral circulation. These vessels are capable of adapting their diameter independently in response to pressure and flow-associated shear stress. Intravasal pressure of about 60 mmHg (e.g. [1]) or higher triggers the development of myogenic tone by the contraction of vascular smooth muscle cells (VSMCs) that are wound around the lumen of small vessels [2]. In all muscle cells, this contraction state depends, among other factors, on the cytosolic Ca^2+^ concentration.

### Ca^2+^ sparks and hyperpolarizing currents in vascular smooth muscle cells

**Figure 1.**
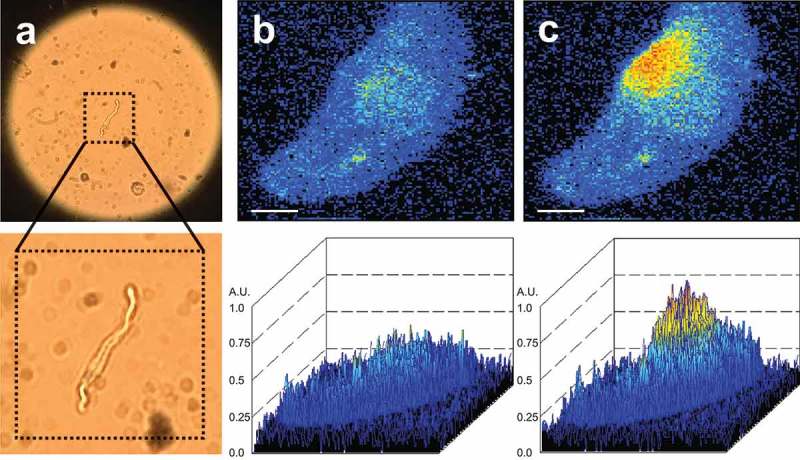
(A) Isolated mesenteric artery smooth muscle cell (VSMC). (B) Ca^2+^ fluorescence image of a Fluo-4-AM–loaded mesenteric VSMC. (C) Ca^2+^ fluorescence image of the same cell as in B during the occurrence of an elementary Ca^2+^ release event (Ca^2+^ spark). The Ca^2+^ fluorescence images were recorded with an inverted microscope (Nikon Eclipse Ti, oil immersion objective x40, numerical aperture 1.3). Details are as described in [6]. Magnification in A (10x ocular, 20x objective, numerical aperture 0.45). Bar, 2 µm.

In vascular smooth muscle cells (VSMCs), the global cytosolic Ca^2+^ concentration is regulated by membrane potential-dependent Ca^2+^ influx pathways, with a major role of Ca_v_1.2 L-type Ca-channels [3–7]. Ca^2+^ sparks are elementary Ca^2+^-release events generated by a single Ca^2+^-release unit (CRU) composed of a cluster of ryanodine receptors (RyRs) in the sarcoplasmic reticulum (SR) [8–10]. Ca^2+^ sparks can be visualized by the use of fluorescent, calcium-sensitive dyes (e.g. Fluo-3/-4/AM) in all types of muscle, including striated and smooth muscle cells (SMCs). The basic properties of Ca^2+^ sparks in human SMCs are similar to arterial SMCs of non-human species [11] . An example of a Ca^2+^ spark recorded in a mouse mesenteric artery SMC is shown in Figure 1.

Ca^2+^ sparks are involved in both positive- and negative-feedback regulation of global cytosolic [Ca^2+^] to regulate the contraction of smooth muscle cells. Unlike Ca^2+^ influx *via* voltage-dependent Ca^2+^ channels (VDCCs), Ca^2+^ release from the SR in the form of Ca^2+^ sparks paradoxically causes vasodilation in peripheral resistance arteries [8,12]. There are two reasons for this counterintuitive effect of Ca^2+^ sparks in arterial vascular smooth muscle cells (VSMCs). First, a single spark produces a remarkably high (10–100 μmol/L) local (∼1% of the cell volume) increase in [Ca^2+^]_i_ [13,14], but increases global [Ca^2+^]_i_ by less than 2 nmol/L [8,10,15]. For triggering Ca^2+^ sparks in VSMCs RyRs need to form clusters [16,17]. At least 10 RyRs open for one Ca^2+^ spark [15,18]. Second, Ca^2+^ sparks occur in close proximity to the cell membrane, where every Ca^2+^ spark activates numerous, approximately 15 [15], large-conductance Ca^2+^-sensitive K^+^ (BK_Ca_) channels, causing K^+^ efflux [8,13,15,19–22]. The resultant “spontaneous transient outward currents” (STOCs) hyperpolarize VSMCs, thereby decreasing Ca^2+^ entry through voltage-dependent Ca^2+^ channels via a deactivation process [15]. The net result of Ca^2+^ spark–BK_Ca_ channel coupling is decreased global [Ca^2+^]_i_ in VSMCs resulting in vasodilation [8,16,19–21,23]. The hallmarks of BK_Ca_ channel biophysics and its relationship with auxiliary subunit expression have been recently reviewed [24]. In some smooth muscle, Ca^2+^ sparks may exert a depolarizing effect through opening Cl^−^ channels [15]. In this regard, TMEM16A Cl^−^ channels have been proposed to comprise an important pathologic mechanism underlying the vasoconstriction and remodeling in pulmonary arteries [25]. TMEM16A is required for peripheral blood vessel contractility and plays a general role in arteriolar and capillary blood flow [26]. TMEM16A is also a key regulator of coronary blood flow and is implicated in the altered contractility of coronary arteries in hypertension [27] (for review see also [28]).

### Excitation-contraction coupling in muscle

**Figure 2.**
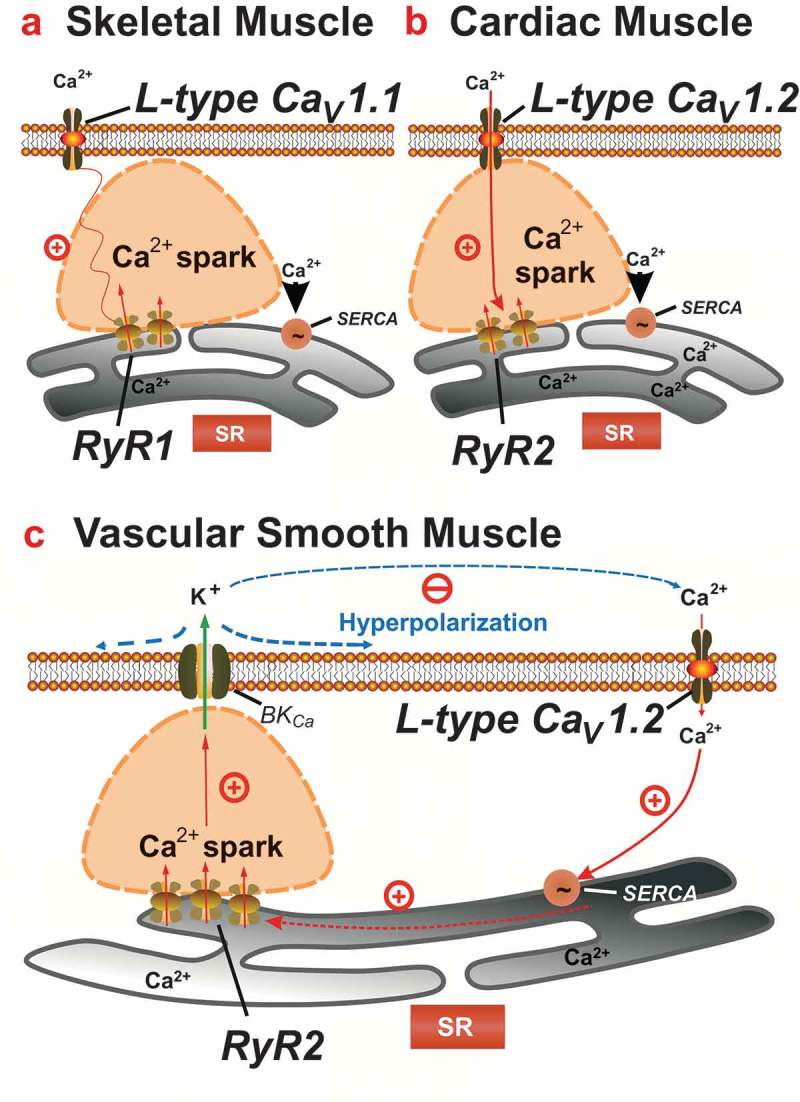
Control of calcium sparks by voltage-dependent calcium channels. (A) Ca^2+^ entry from extracellular space is not required for calcium release in skeletal muscle. Charge movement within L-type channel Ca_v_1.1 activates RyR1 channel via a direct physical interaction. Ca^2+^ efflux from SR through the opened RyR1 channel activates nearby RyR (RyR1 and RyR3) channels *via* calcium-induced calcium release (CICR). (B) In cardiomyocytes Ca_v_1.2 mediates influx of extracellular Ca^2+^ into cytosol. Ca^2+^ then binds to and activates RyR2 channels *via* CICR. (C) Triggering of Ca^2+^ sparks is not controlled by rapid, direct cross-talk between Ca_v_1.2 channels and RyR2 in arterial smooth muscle in contrast to cardiac and skeletal muscle cells. Instead, Ca_v_1.2 channels contribute to global cytosolic [Ca^2+^], which in turn influences luminal SR calcium and thus calcium sparks. SERCA: sarco/endoplasmic reticulum calcium ATPase, BK: big conductance calcium-activated K^+^ channel. Modified from [29].

In different muscle types, there are unique mechanisms of excitation-contraction coupling to regulate the cytosolic Ca^2+^ concentration [29] (Figure 2). *In skeletal muscle*, a direct physical interaction between the L-type channel Ca_v_1.1 and predominantly type 1 ryanodine receptors (RyR1) forms the basis of excitation contraction (E-C) coupling [29]. In this respect, opening of Ca_v_1.1 channel upon membrane depolarization acts as a voltage sensor with conformational changes of the channel leading to a physical interaction with RyR1, promoting its opening, and triggering the release of Ca^2+^ from internal stores (Figure 2(A)) [29–31]. Thus, in skeletal muscle, Ca^2+^ entry is not required for RyR mediated calcium release [32]. *In cardiac muscle*, physical coupling between Ca_v_1.2 L-type calcium channels and RyRs is not involved in E-C coupling. Instead, membrane depolarization of cardiac myocytes leads to the opening of the L-type channels with Ca^2+^ influx stimulating the opening of the RyR (predominantly RyR2) and subsequent Ca^2+^ release from SR^2^ [9,33–35] (Figure 2(B)). The close proximity between SR and plasma membrane (t-tubuli) is the morphological requisite for effective calcium-induced calcium release (CICR) in cardiac cells [29].

*In arterial smooth muscle*, a prerequisite for generation of Ca^2+^ sparks is a close proximity of RyRs, BK_Ca_ channels, and VDCCs, forming a functional unit (Figure 2(C)) [15]. Parts of the smooth muscle SR membrane are less than 20 nm distant from the plasma membrane [36]. RyR channels have been localized to both the peripheral and central SR [16,36,37]. VDCC and RyR channels colocalize in smooth muscle [16,38].

The ratio of RyRs/L-type channels in skeletal muscle is about 0.5–0.9 [39]. The RyRs/L-type channel ratio in cardiomyocytes is higher and is 3.7 in rabbits, >10 in ferrets and 4 to 7.3 in rats [39,40]. The amount of RyRs forming individual calcium release units (CRUs) in cardiomyocytes seems to vary (up to >100) with a distribution described by exponential equations [41]. The RyRs/L-type channel ratio in bladder SMCs was determined as 2:1 [42].

### Ryanodine receptors

RyRs are major Ca^2+^-release channels in the SR membrane of myocytes that contribute to the regulation of contractility. They are large proteins (~565 kDa), regulated by a multitude of factors and conditions like pH, phosphorylation, oxidation, inorganic ions like Ca^2+^ and Mg^2+^, the name giving ryanodine, caffeine, nucleotides, neomycin, tetracaine, halothane, isoflurane, dantrolene, imperatoxin, arachidonic acid and many others (for review see, e.g. [43–45]). Boittin et al. [46] found RyRs in SR sections close to the cell membrane. Fritz et al. [47] confirmed that finding for RyR1 in urinary bladder SMCs. However, they detected RyR2 and RyR3 mainly in the deep SR. Although already expressed at birth, RyRs form clusters during ontogeny of arterial smooth muscle cells. These CRUs are a prerequisite to produce Ca^2+^ sparks as elementary Ca^2+^ release events in VSMCs to produce vasodilation in resistance arteries [16].

The ability of drugs to regulate RyRs to generate Ca^2+^-sparks in VSMCs (portal vein) was demonstrated for caffeine (1 mmol/L) or low doses of ryanodine (1 µmol/L) [48]. VSMCs exhibit all three known RyR isoforms (RyR1, −2, −3). The impact of individual RyR isoforms on elementary Ca^2+^ signaling and adaptive vascular responses has been clarified in the last years with different approaches including silencing single isoforms by antisense (as) oligonucleotides (e.g.[49]) and an inducible smooth muscle-specific RyR2 knock-out mouse model [6].

**Figure 3.**
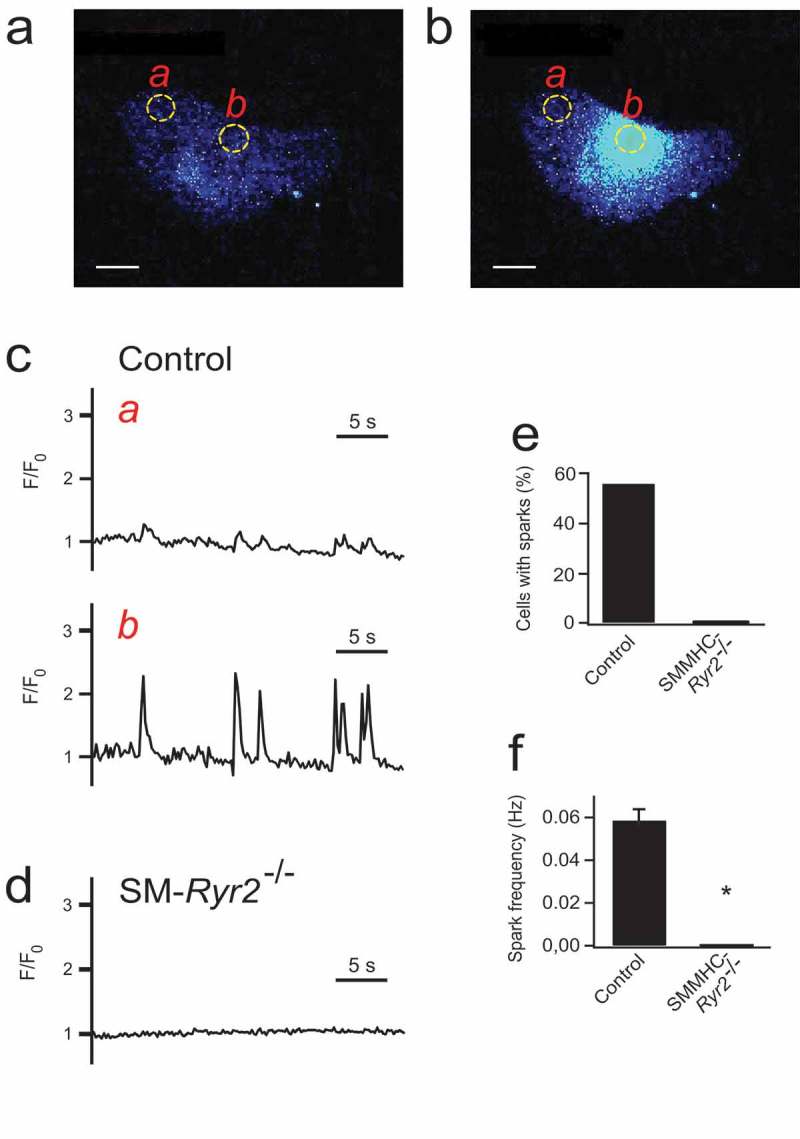
Ca^2+^ sparks in wild-type and smooth muscle myosin heavy chain (SMMHC)–*Ryr*2^−/-^ tibial artery smooth muscle cells (SMCs). (A) Ca^2+^ fluorescence image of a Fluo-4-AM–loaded control SMC. (B) Ca^2+^ fluorescence image of the same cell as in A during the occurrence of a Ca^2+^ spark. Two-dimensional images were recorded at a rate of 5/s using an inverted microscope (Nikon Eclipse Ti, oil immersion objective x40, numerical aperture 1.3;) as described in [6]. (C) Time course of Ca^2+^ fluorescence changes in cellular regions of interest (ROIs) without sparks (ROI a, left) and with sparks (ROI b, right). (D) Time course of Ca^2+^ fluorescence changes in an ROI (similar size as that in A) of a SMMHC-*Ryr*2^−/-^ SMC. (E) Percentage of SMCs with Ca^2+^ sparks. (F) Ca^2+^ spark frequencies in SMCs from control and SMMHC-*Ryr*2^−/-^ mice. F/F0, fluorescence/background fluorescence. Bar, 2 µm. *P < 0.05. Modified from [6].

Coussin et al. reported that treatment with antisense oligonucleotides against RyR1, the dominant RyR subform in skeletal muscle [50], and treatment with antisense oligonucleotides against RyR2, the dominant RyR subform in heart [50,51], abolished Ca^2+^ sparks in rat portal vein myocytes [49]. The authors propose the existence of mixed Ca^2+^ channel CRUs with both receptor subtypes necessary to function. Responses to caffeine were weakened, but not completely abolished, with RyR2 or RyR1 silenced [49]. Essin & Gollasch [29] observed that *in vivo* suppression of RyR2 expression by antisense oligonucleotides increases the SR Ca^2+^ content without effects on spontaneous calcium sparks in rat cerebral arteries. Noteworthy, the frequency and amplitude of Ca^2+^ sparks in cerebral arteries was reported to depend on the size of RyR2 clusters [52], which supports the idea the RyR2 could play a dominant role in Ca^2+^ spark generation in arterial smooth muscle. Very recent data demonstrated that conditional smooth muscle-specific deficiency of RyR2 in mice leads to complete loss of SR Ca^2+^ release and contractile reactivity following caffeine application of various arteries [6]. RyR2 has an essential function in systemic blood pressure control and vascular adaptive responses to pressure. In the conditional SM-specific RyR2 knock-out mouse model, the ability of the VSMCs to produce Ca^2+^ sparks was completely abolished by lack of RyR2 (Figure 3). Furthermore, the negative feedback control of myogenic tone was demonstrated to rely on functional RyR2 [6]. Functional RyR2 also limited the pressure increase in the chronic phase of hypoxic pulmonary vasoconstriction [6,53]. In contrast, RyR2 do not play a role in the adaptive vascular response to blood flow after a femoral artery ligation [6,54].

RyR3 are widely expressed throughout different organs of the body, including VSMCs and other smooth muscle types (e.g. in digestive tract and uterus) [50,51]. The physiological data concerning Ca^2+^ signaling are diverse and in part contradictory. Silencing RyR3 with antisense oligonucleotides in rat portal vein myocytes reduced the Ca^2+^ responses to caffeine or phenylephrine in a solution containing 10 mmol/L Ca^2+^; thus, RyR3 might be activatable by local high [Ca^2+^][55]. However, spontaneous Ca^2+^ sparks were not affected in this study. In mouse cerebral artery VSMCs of *Ryr*3^−/-^ mice [56], the frequencies of Ca^2+^ sparks and STOCs were increased [57], indicating a possible influence of RyR3 on Ca_v_1.2/RyR2 coupling in arterial smooth muscle. A possible reason could be a lower sensitivity of RyR3 to Ca^2+^ dependent inactivation than that of RyR2 [58]. The RyR3-mediated prolonged Ca^2+^ release would inactivate RyR2 channels [29]. A specific role of RyR3 in calcium spark signaling in non-spiking smooth muscle compared to spiking smooth muscle may be caused by different expression levels [59] and/or splicing variants of RyR3 [60,61]. A dominant negative (DN) variant has been proposed to function as predominant RyR3 variant in mesenteric smooth artery muscle [62]. The authors showed that RyR3 (especially DN-RYR3) suppressed the Ca^2+^ release by forming complexes with RyR2.

BK_Ca_ channels activated by Ca^2+^ sparks appeared to hyperpolarize and dilate pressurized myogenic arteries because ryanodine and thapsigargin depolarized and constricted these arteries to an extent similar to that produced by blockers of BK_Ca_ channels [8,12]. Pressure-induced myogenic tone is involved in autoregulation of local blood flow and confers protection against excessive pressure levels in small peripheral arteries and arterioles. In a number of arteries, the development of myogenic tone relies on ligand-independent mechanoactivation of angiotensin II type 1a receptors [1], a G_q/11_-protein coupled receptor type [63]. Interestingly, angiotensin II has been found to stimulate Ca^2+^ sparks *via* AT_1_ receptors and L-type Ca^2+^ channels [64]. This pathway may cause increased Ca^2+^ spark–BK_Ca_ channel coupling during ligand-dependent vasoconstriction to limit excess pressure levels. However, the molecular mechanism is not entirely clear since activators of protein kinase C have been found to decrease Ca^2+^ spark frequency in smooth muscle cells from cerebral arteries [65]. In addition to RyRs, inositol 1,4,5-trisphosphate (IP_3_) receptors may have a function in regulating the cytosolic Ca^2+^ concentration, since unitary Ca^2+^ release may also occur due to inositol trisphosphate receptor (IP_3_R) activation in the SR at the same intracellular Ca^2+^ reservoir as ryanodine receptors [46,66–68]. IP_3_ dynamically regulates assembly of IP_3_ receptors into clusters that underlie the hierarchical recruitment of elementary IP_3_ receptor-mediated Ca^2+^ release events (Ca^2+^ puffs) [69]. Interestingly, opening of both RyR1 and IP_3_ receptors can be allosterically coupled to a displacement of the N-terminal domain from the following two domains. This displacement can be affected by glutathionylation of a highly reactive cysteine residue, or ligand binding [70]. In human coronary artery VSMCs, Ca^2+^ entry into the cell through reverse-mode Na^+^/Ca^2+^ exchange determines calcium store refilling, which in turn regulates STOC generation [71,72].

RyRs change their physiological function during the aging process. Although there are no studies on the effects of age on Ca^2+^ sparks in arterial smooth muscle, age-dependent alterations in RyR function influence the node cycle length and atrio-His interval in hearts of rats [73]. RyRs may modulate gap junction formation during stress and aging. The pan RyR activator caffeine decreased the expression of gap junction forming proteins, the connexins Cx40, Cx43, and Cx45, and reduced the contractility of cardiomyocytes [74]. These effects have been attributed to long-lasting activated ryanodine receptors by caffeine. With aging, expression of Cx40, SERCA type 2a, and the L-type Ca^2+^ channel Ca_v_1.3 increases while the expression of Cx43 and RyR2 decreases in certain parts of the heart, such as the inferior nodal extension and proximal penetrating bundle [73]. Gap junction impairment could also disturb cell-cell communications between VSMCs necessary for vascular adaptation to pressure and local blood flow control [75].

## Ca^2+^ channels in vascular smooth muscle cells

Voltage-dependent Ca^2+^ channels in the plasma membrane of VSMCs are regulated by membrane potential. They dominate the extracellular Ca^2+^ influx into VSMCs in response to transmural pressure to elevate the global cytosolic [Ca^2+^] to cause myogenic vasoconstriction [10,76]. Ca^2+^ influx through Ca^2+^ channels contributes to global cytosolic [Ca^2+^], which in turn also influences luminal SR calcium and triggers Ca^2+^ sparks to activate BK_Ca_ channels. The resultant BK_Ca_ channel mediated plasma membrane hyperpolarization, which opposes myogenic vasoconstriction, i.e. Ca^2+^ sparks cause paradoxically arterial vasodilatation [8,13–15,29]. In addition to VDCCs, transient receptor potential (TRP) channels and ionotropic purinergic (P2X) receptors, which are nonselective cation channels, also contribute to extracellular Ca^2+^ influx [77–79].

VDCCs are multi-subunit complexes comprising a pore-forming α1 subunit and regulatory β, α2δ, and γ subunits [80]. Electrophysiological studies performed with vertebrates VDCC subunits have demonstrated that the α1 subunit largely determines both the pharmacological and biophysical properties of Ca^2+^ currents [7,81]. These properties have defined different current subtypes, according to their Low or High Voltage for Activation (LVA or HVA), their Long-lasting or Transient kinetics of inactivation (L or T-type), and their distinct sensitivities to Dihydropyridines (DHP) and different toxins. VDCCs can be grouped in three major families based on their α1 subunit amino acid sequence similarity: Ca_v_1, Ca_v_2, and Ca_v_3. VDCCs have been differentiated into T-(transient), N-(originally termed as neither T nor L [82]) and L-(long lasting) channels. N-channels are apparently present only in neuronal cells whereas T-type and L-type channels have been identified in most cells, including vascular smooth muscle cells (see Table 1).

**Table 1.** Voltage-dependent Ca^2+^ channels (VDCCs) expressed in vascular smooth muscle.

α1 Subunit	Localization	Reference
L-type Ca_v_1.2	(vascular) smooth muscle, cardiac muscle, neurons, endocrine cells	[7,83–88]
L-type Ca_v_1.3	sensory cells, endocrine cells, neurons, low density in heart and vascular smooth muscle	[85,89]
T-type Ca_v_3.1	(vascular) smooth muscle, cardiac muscle, neurons	[83–86]
T-type Ca_v_3.2	(vascular) smooth muscle, cardiac muscle, neurons, kidney, liver	[83–86]

## L-type Ca^2+^ channels in vasculature

Voltage-dependent L-type Ca^2+^ channels play a critical role in excitation-contraction coupling through calcium signaling in cardiac, skeletal, and smooth muscle. They have a large single-channel conductance compared to T-type channels, are sensitive to dihydropyridine blockers, and encoded by the Ca_v_1 family of α1 subunits[85]. Four subtypes of L-type Ca^2+^ channels (Ca_v_1.1–1.4) have been identified in cardiac, smooth muscle and neurons. They are of particular functional importance and essential for cardiac contraction and for neuronal function [90,91]. L-type Ca_v_1.2 channels provide an important pathway for influx of extracellular Ca^2+^ and their graded activation plays a central role in setting cytosolic [Ca^2+^] in smooth muscle contraction [15,92].

Ca_v_1.2 channels have been explored in vascular smooth muscle by pharmacological or genetic tools, and they exhibit a critical role in the regulation of Ca^2+^ sparks, STOCs, and myogenic tone in arteries [3,5,7]. Deletion of the smooth muscle-specific Ca_v_1.2 channel gene in (SMAKO) mice decreased Ca^2+^ spark frequency by ~70-80%, and the STOCs frequency by ~50% [3,5]. Resistance arteries from SMAKO mice show very weak myogenic constriction in response to increased intravascular pressure [7,93].

Ca_v_1.3, encoded by the CACNA1D gene, is expressed together with Ca_v_1.2 in many tissues, including neurons, endocrine cells, as well as smooth muscle [94]. Although Ca_v_1.3 can mediate Ca^2+^ sparklets in vascular smooth muscle, Ca_v_1.2 channels underlie predominant Ca^2+^ currents, which were similar to those recorded from cells lacking Ca_v_1.3 channels [95].

## T-type Ca^2+^ channels in vasculature

Ca_v_3.1 and Ca_v_3.2 T-type channels have been found in rat and human renal microvessels (preglomerular vessels, juxtamedullary efferent arterioles, and vasa recta) to contribute to control of blood pressure, glomerular filtration rate, and salt and water homeostasis [86,96,97]. Ca_v_3.1 and Ca_v_3.2 channels have been identified by RT-PCR, Western blot, immunolocalization or functional studies in cerebral arteries [84,98,99], aorta and mesenteric arteries of rats [100,101], in human subcutaneous vessels [101] and in mouse cremaster arterioles [102]. T-type channels could contribute to alterations of rat cerebral network perfusion (20-50% of the total response) as biophysical modeling revealed based on data obtained in arterial diameter measurements [84].

## Role of VDCC-RyR signaling on Ca^2+^ spark generation in vascular smooth muscle

Similar to the heart, L-type Ca_v_1.2 Ca^2+^ channels are presumably the predominant pathway by which extracellular Ca^2+^ triggers Ca^2+^ sparks in arterial smooth muscle. In cardiac muscle cells, tight coupling between Ca_v_1.2/RyR2 seems to underlie CICR to generate Ca^2+^ sparks. Wang et al. [103] determined the coupling fidelity of L-type Ca^2+^ channels and RyR in rat ventricular myocytes with 0.71, i.e. 71% opening events of L-type channels trigger Ca^2+^ sparks. In contrast, RyRs activation is not tightly linked to the opening of L-type Ca^2+^ channels to trigger Ca^2+^ sparks in smooth muscle [3,104]. The analysis of latencies of calcium sparks appearing after L-type Ca_v_1.2 channel openings at different membrane potentials [3,104] revealed a rather loose coupling. Nevertheless, Ca_v_1.2 channels could be shown to contribute to global cytosolic [Ca^2+^], which in turn influences luminal SR calcium and thus Ca^2+^ sparks in arterial smooth muscle [3,105]. The loose coupling between L-type Ca_v_1.2 channel and RyRs is presumably due to an increase in the effective distance between both channels, resulting in an uncoupling of the obligate relationship that exists in striated muscle between the action potential and calcium release [104].

The close proximity between Ca_v_ channels and RyRs for E-C coupling in striated muscle is ensured by placing the L-type Ca_v_1 channels inside t-tubules precisely aligned to parts of the SR in skeletal muscle [106-108] and cardiomyocytes [90,109,110]. Also in VSMCs a precise delivery of the Ca^2+^ ions supplied by VDCCs with short diffusion ways to the SR is essential [111] to trigger Ca^2+^ sparks for the indirect regulation of the VSMC contraction state. However, smooth muscle cells do not have t-tubuli. Instead, they possess abundant cell membrane caveolae [112]. Löhn et al. were the first to propose that caveolae could represent additional structural elements necessary for the generation of Ca^2+^ sparks in cardiomyocytes and arterial smooth muscle [111]. This concept is supported by findings of Boittin et al. [46], who found RyRs in vicinity of caveolae. Impairments of the caveolae structure, e.g. via cholesterol depletion with methyl-beta-cyclodextrin or by genetically knocking out essential proteins like EHD2 or caveolin 1, reduce the frequency of Ca^2+^ sparks [4,5] (Fan et al. 2019 (submitted)). We recently also found that BK_Ca_ channels are not the downstream mediators of perivascular adipose tissue-dependent arterial vasorelaxation [113–115].

## Caveolae microdomains and Ca^2+^ sparks

**Figure 4.**
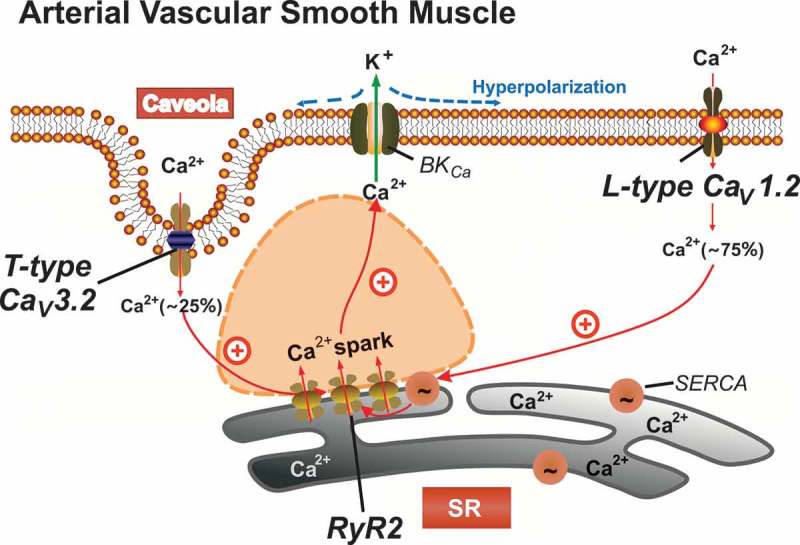
Proposed model of the role of Ca_v_1.2 and Ca_v_3.2 channels in arterial smooth muscle Ca^2+^ spark generation. Ca^2+^ sparks are produced by opening of clustered ryanodine receptors (RyR2) in the SR, which produces a negative-feedback effect on vasoconstriction. This vasodilatory effect is mediated by activation of large-conductance Ca^2+^-activated K^+^ (BKCa) channels, which results in hyperpolarization of VSMCs and reduced global cytosolic [Ca^2+^]. The majority (~70-80%) of Ca^2+^ sparks are triggered by Ca_v_1.2 channels contributing to global cytosolic [Ca^2+^], which in turn influences luminal SR calcium via SERCA [3,104]. Ca_v_3.2 T-type channels contribute to a minor extent [5]. SERCA, calcium pump; SR, sarcoplasmic reticulum; VSMC, mesenteric artery vascular smooth muscle cell.

L-type Ca_v_1.2 is typically thought to play a predominant role in the signaling pathway of VDCCs-RyRs-BK_Ca_ in vascular smooth muscle [3]. Nevertheless, pharmacological or genetic Ca_v_1.2 channel ablation does not fully eliminate Ca^2+^ spark events and STOCs, RyR2-mediated Ca^2+^ spark-dependent regulation of arterial myogenic tone [3,98,103]. L-type Ca_v_1.2 channels are not the only voltage-dependent calcium channels expressed in vascular tissue (see Table 1). T-type Ca_v_3.2 channels reside inside caveolae [111,112]. In mouse mesenteric arteries, but not in tibial or cerebral arteries, they represent additional Ca^2+^ influx channels to trigger Ca^2+^ sparks [4,5,112] (Figure 4).

T-type calcium channels were originally described as low-voltage-activated (LVA) channels because they can be activated at more negative membrane potentials compared to high-voltage-activated channels, such as Ca_v_1.2. They are also characterized by their tiny and transient inward Ca^2+^ currents (called T-type currents) resulting from small depolarization [116,117]. No specific antagonists are available to separate each subtype of T-type channel. Mibefradil, the most commonly used T-type channel blocker, has not only equal function on each of the three T-type channel subtypes [118], but also has been shown to block other ion channels [119–121]. Low concentrations of nickel (with an IC_50_ value of about 13 µmol/L) have been demonstrated successfully in Ca_v_3.2 channel blocking, whereas ~20-fold of this IC_50_ concentration was necessary to inhibit Ca_v_3.1 channels or L-type channels, with IC_50_ values of 250 µmol/L and 324 µmol/L, respectively [122,123]. Experiments using Ca_v_3.1 knockout mice indicate that Ca_v_3.1 channels contribute to the sinoatrial node pacemaker activity and atrioventricular conduction but do not control systemic blood pressure [124]. Also, Ca_v_3.1 channels appear to be necessary for proper progression of the cell cycle in human pulmonary artery smooth muscle cells [125]. However, there are no reports on vascular abnormalities in Ca_v_3.1 knock-out animals.

Research based on the pharmacological (Ni^2+^) or/and genetic tools clearly ascertained the contribution of Ca_v_3.2 in VSMCs. Findings reveal that Ca^2+^ influx through Ca_v_3.2 channel triggers Ca^2+^ spark generation and then activates BK_Ca_ channels to hyperpolarize and relax certain artery types, such as cerebral and mesenteric arteries [112,126].

Although knockout mice provide an important and reliable tool for investigation of the roles of Ca_v_3.1 and Ca_v_3.2 channels, the potential of compensatory and masking effects (or interference) caused by L-type channels remains a general challenge in specific T-type deletion studies. In this state-of-affairs, smooth muscle-specific Ca_v_1.2^-/-^ (SMAKO) mice [3] have been successfully used to explore the role of T-type Ca^2+^ channels in vascular function. Intriguingly, the Ca_v_3.2 blocker nickel fully abolished Ca^2+^ sparks after silencing Ca_v_1.2 channels in mouse mesenteric smooth muscle cells. However, nickel failed to inhibit Ca^2+^ sparks in mouse tibial and cerebral arteries. Thus, T-type channels provide sufficient Ca^2+^ influx to trigger Ca^2+^ sparks (responsible for about 20%~30%) at the resting membrane potential in certain artery types, when the interference from L-type Ca_v_1.2 channels is excluded (Figure 4) [5]. T-type Ca_v_3 channel activity on vascular tone regulation is most prominent at voltages close to the VSMC resting potential [112] and at low intravasal pressures (40–80 mmHg) [127,128].

Based on these data, the question was raised whether direct *vs*. indirect Ca_v_3.2 channel-RyR2 coupling underlies Ca^2+^ spark generation, for example in specialized microdomains, such as caveolae. Recent data show that there is close apposition of Ca_v_3.2 channels in caveolae to RyRs, which is crucial for T-type Ca_v_3.2 to generate Ca^2+^ sparks. The effective distance between Ca_v_3.2 and RyRs in a restricted microdomain is ~15 nm between caveolae in the plasma membrane and the sarcoplasmic reticulum [112]. Unfolding the pits structure of caveolae, pharmacologically or genetically, blocked the Ni^2+^-sensitive Ca^2+^ sparks and Ca_v_3.2/BK_Ca_-mediated feedback in vascular smooth muscle [4,5,112].

Interestingly, a novel junctophilin–caveolin interaction has been identified in mesenteric arteries, which enables efficient coupling between RyRs and BK_Ca_ channels in the VSMC Ca^2+^ microdomain. The data indicate that junctophilin-2, interacting with the caveolae-forming protein caveolin-1, generates bridges between the plasma membrane and the SR and thereby contributes to the formation of microdomains essential for Ca^2+^ sparks. Downregulation of junctophilin-2 diminished the amplitude of STOCs. The frequency of Ca^2+^ sparks was increased but not the STOCs frequency [129]. The authors conclude that junctophilin-2/Cav1 interaction provides a structural and functional basis for the Ca^2+^ microdomain at plasma membrane-SR junctions and mediates cross-talk between RyRs and BK_Ca_ channels, converts local Ca^2+^ sparks into membrane hyperpolarization, and contributes to stabilizing resting tone in VSMCs.

## Perspectives

In the past decade, a number of new molecular mechanisms have been identified which control Ca^2+^ spark–BK_Ca_ channel coupling and relaxation in arterial smooth muscle. As such, very recent data identified major roles of RyR2, Ca_v_3.2 T-type Ca^2+^ channels and caveolae in these processes. These molecular components and associated pathways may represent novel promising molecular targets for the development of new drugs to treat high blood pressure and regional blood flow. They could be potentially important for understanding BK_Ca_ channel-mediated pathways in human baroreflex control [130]. Further work is necessary to study the changes these molecules undergo during aging. Recent evidence also supports the reverse role for BK_Ca_ channels, in which they facilitate Ca^2+^ influx into the cell interior through open nonselective cation (e.g., transient receptor potential; TRP) channels in accord with robust electrical (hyperpolarization) and concentration (~20,000-fold) transmembrane gradients for Ca^2+^ [131]. Such an arrangement supports a feed-forward activation of membrane hyperpolarization while potentially affecting Ca^2+^ spark–BK_Ca_ channel coupling. It is tempting to speculate that TRP channels might represent promising new targets in chronic, age-related cardiovascular disease to affect relaxation of arterial smooth muscle by Ca^2+^ sparks. Further work is also required to ascertain the precise relationship between Ca_v_3.2 mediated Ca^2+^ influx and Ca^2+^ sparks, and the contribution of Ca_v_3.2 channels on luminal calcium content. We proposed experimental study designs for distinguishing between biological and technical replicates in such studies using isolated arteries [132]. Future work is also required to study the role of these pathways in aging and cardiovascular disease states. In light of the global obesity epidemic, it will be important to study the impact of adipokins, such as ADRF, released from perivascular adipose tissue [133] on arterial Ca^2+^ spark–BK_Ca_ channel coupling and relaxation and their role in the treatment and prevention of chronic, age-related cardiovascular and metabolic diseases.
